# 2-Fluoro­benzyl (*Z*)-2-(5-chloro-2-oxoindolin-3-yl­idene)hydrazine-1-carbodi­thio­ate dimethyl sulfoxide monosolvate

**DOI:** 10.1107/S2414314625008491

**Published:** 2025-10-03

**Authors:** Aidan P. McKay, David B. Cordes, Mohd Abdul Fatah Abdul Manan

**Affiliations:** a EaStCHEM School of Chemistry, University of St Andrews, St Andrews, Fife KY16 9ST, United Kingdom; b Faculty of Applied Sciences, Universiti Teknologi MARA, 40450 Shah Alam, Selangor, Malaysia; University of Aberdeen, United Kingdom

**Keywords:** crystal structure, hydrogen bonding, halogen bonding, chalcogen bonding

## Abstract

In the title solvate, C_16_H_11_ClFN_3_OS_2_·C_2_H_6_OS, the C=N bond adopts a *Z*-configuration facilitating the formation of an intra­molecular N—H⋯O hydrogen bond to the carbonyl O atom in an *S*(6) loop. The di­methyl­sulfoxide solvent mol­ecule accepts a strong discrete N—H⋯O hydrogen bond from the γ-lactam grouping. In the extended structure, C_ar_—H⋯S hydrogen bonds and quasi-Type I/II Cl⋯F halogen⋯halogen bonds are observed, while adjacent dimethyl sulfoxide mol­ecules form S⋯O chalcogen-bonded chains.

## Abstract 2

The packing of the title solvate features hetero-halogen⋯halogen (Cl⋯F), and chalcogen (S⋯O) bonding, as well as non-standard offset π–π stacking.

## Graphical abstract

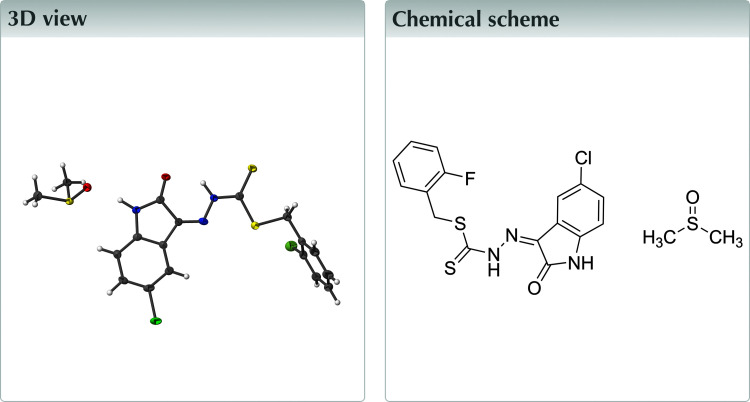

## Structure description

Halogen bonding is defined as a directional non-covalent attractive inter­action between an electrophilic region of a halogen atom (the halogen-bond donor) and a Lewis base (the acceptor) (Desiraju *et al.*, 2013 ▸). The halogen⋯halogen subset of halogen bonding is divided into four major categories based on their geometry (Saha *et al.*, 2023 ▸) with the first two being Type I (90 < *θ*_1_ ≃ *θ*_2_ < 180°) and Type II (*θ*_1_ ≃ 180°, *θ*_2_ ≃ 90°), where *θ*_1_ and *θ*_2_ are the C—*X*⋯*X*′ and C—*X*′⋯*X* angles, respectively (*X*, *X*′ = F, Cl, Br, I; Desiraju & Parthasarathy, 1989 ▸; Nayak *et al.*, 2011 ▸). Organic mol­ecules containing fluorine are of inter­est due to their prevalence in pharmaceuticals (Inoue *et al.* 2020 ▸; Du *et al.* 2025 ▸), with F atoms shown to act as the nucleophilic acceptors in a range of inter­molecular inter­actions including halogen and chalcogen bonding (Cole & Taylor, 2022 ▸), as well as unusual short C—F⋯F—C inter­actions (Singla *et al.*, 2023 ▸), while having a van der Waals radii not much larger than that of hydrogen. As part of our studies in this area, we now report the synthesis and structure of the title solvate, C_16_H_11_ClFN_3_OS_2_·C_2_H_6_OS (**1**).

**Figure 1:**
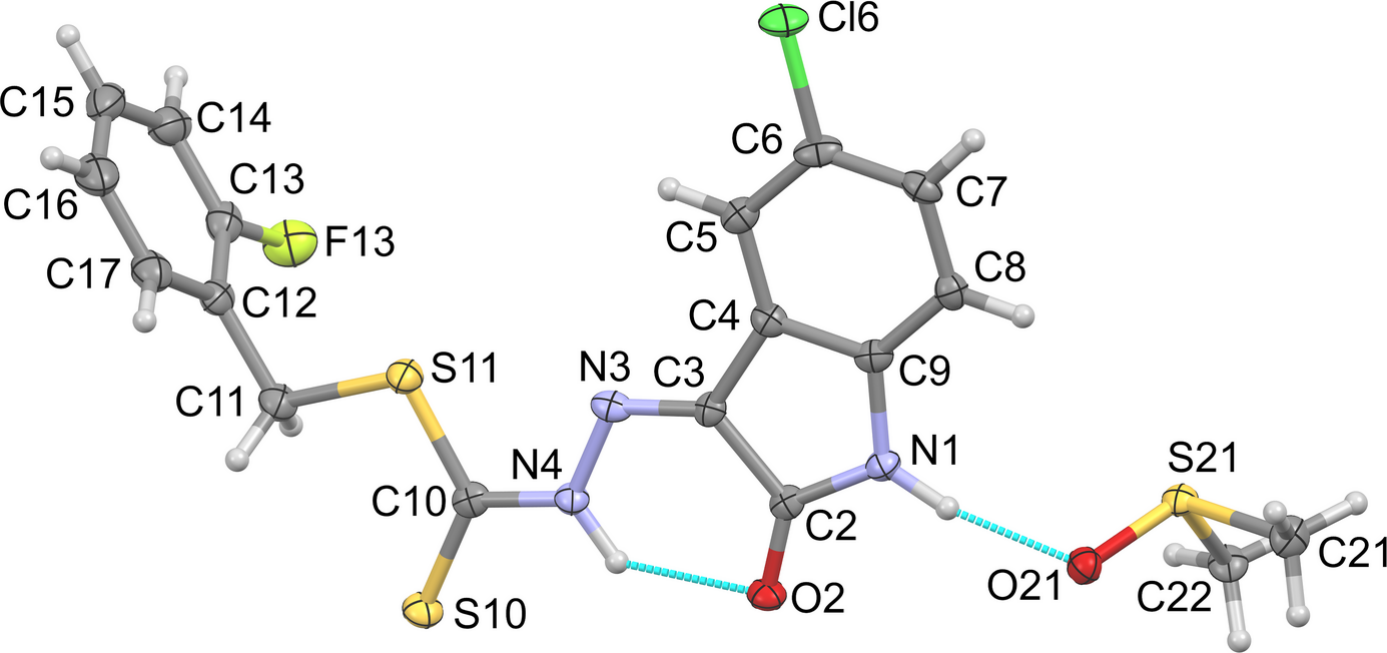
The mol­ecular structure of **1** with displacement ellipsoids drawn at 50% probability and hydrogen bonds shown as blue dashed lines.

**Figure 2:**
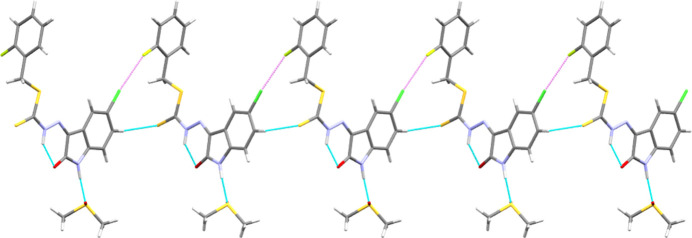
View along the *b* axis showing the packing of **1** into chains along [001] through a mixture of non-classical hydrogen bonding (blue dashed lines) and halogen bonding (violet dashed lines).

Compound **1** crystallizes in the monoclinic space group *P*2_1_/*c* with one mol­ecule and a di­methyl­sulfoxide (DMSO) solvent mol­ecule in the asymmetric unit (Fig. 1 ▸). The C=N bond displays a *Z*-configuration, resulting in the hydrazine N4—H hydrogen atom being directed towards the isatin-O2 atom giving a intra­molecular N—H⋯O hydrogen bond, generating an *S*(6) ring motif, while the N1—H amide of the γ-lactam forms a discrete N—H⋯O hydrogen bond to the DMSO solvate (Table 1 ▸). The isatin (O2) group is *syn* to the thione S10 atom with the *S*-2-fluoro­benzyl moiety orientated in the opposite direction. The structure shows a small bow between the methyl­idenehydrazinecarbodi­thio­ate (MHT) grouping and the γ-lactam ring of 6.52 (12)° and the 2-fluoro­phenyl ring is twisted perpendicular to the MHT at 89.67 (11)°. Non-classical C_ar_—H⋯S hydrogen bonds from the fused aromatic ring (C7) of the isatin moiety to S10 of the adjacent mol­ecule related by the glide plane (*x*, −*y* + 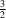, *z* + 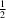) link mol­ecules into pleated *C*(10) chains propagating along [001]. Alongside this hydrogen bond, there is a Cl⋯F halogen⋯halogen bond [Cl6⋯F13 = 2.936 (3) Å, C6—Cl6⋯F13 = 171.08 (15)°, C13—F13⋯Cl6 = 147.1 (2)°] between the same adjacent mol­ecules, which adopts a quasi-Type I/II geometry [Δθ = 24.0 (4)°, where Δθ = |θ_1_ – θ_2_|; Tothadi *et al.*, 2013 ▸] (Fig. 2 ▸). Hetero-halogen⋯halogen inter­actions (*X* ≠ *X*′) have been found to generally favour Type II inter­actions (30 < Δθ < 90°), which have attractive character, in the same electrophile⋯Lewis base manner as hydrogen bonding (Veluthaparambath *et al.*, 2023 ▸), although Cl⋯F halogen bonds generally show a spread of types more similar to homo-halogen⋯halogen inter­actions (Pedireddi *et al.*, 1994 ▸). The observed quasi-Type I/II Cl⋯F halogen bond is consistent with the general trend of Type II hetero-halogen⋯halogen inter­actions where θ for the heavier halogen is greater than θ at the lighter atom (Tothadi *et al.*, 2013 ▸). The pleated chains further pack together through weak, non-standard, π–π stacking [centroid_C4>C9_⋯centroid_N3=C3_ = 3.302 (4) Å] between the fused benzo ring (C4–C9), of the isatin moiety and the C3=N3 bond of a translation-related (*x*, *y* + 1, *z*) adjacent mol­ecule. Concurrently, translation-related (*x*, *y*±1, *z*) DMSO solvent mol­ecules form chalcogen-bonded [S21⋯O21 = 3.209 (3) Å, S21—O21⋯S21 = 173.82 (14)°] chains along [010], which, together with the C_ar_—H⋯S hydrogen bonds and the π–π stacking, results in the formation of sheets parallel to (100). These sheets then pack together through a variety of weaker C—H⋯F [H⋯F = 2.865 (3) Å, C⋯F = 3.761 (5) Å] and C—H⋯Cl [H⋯Cl = 2.9968 (10)–3.3158 (10) Å, C⋯Cl 3.567 (4)–4.080 (4) Å] inter­actions.

**Figure 3:**
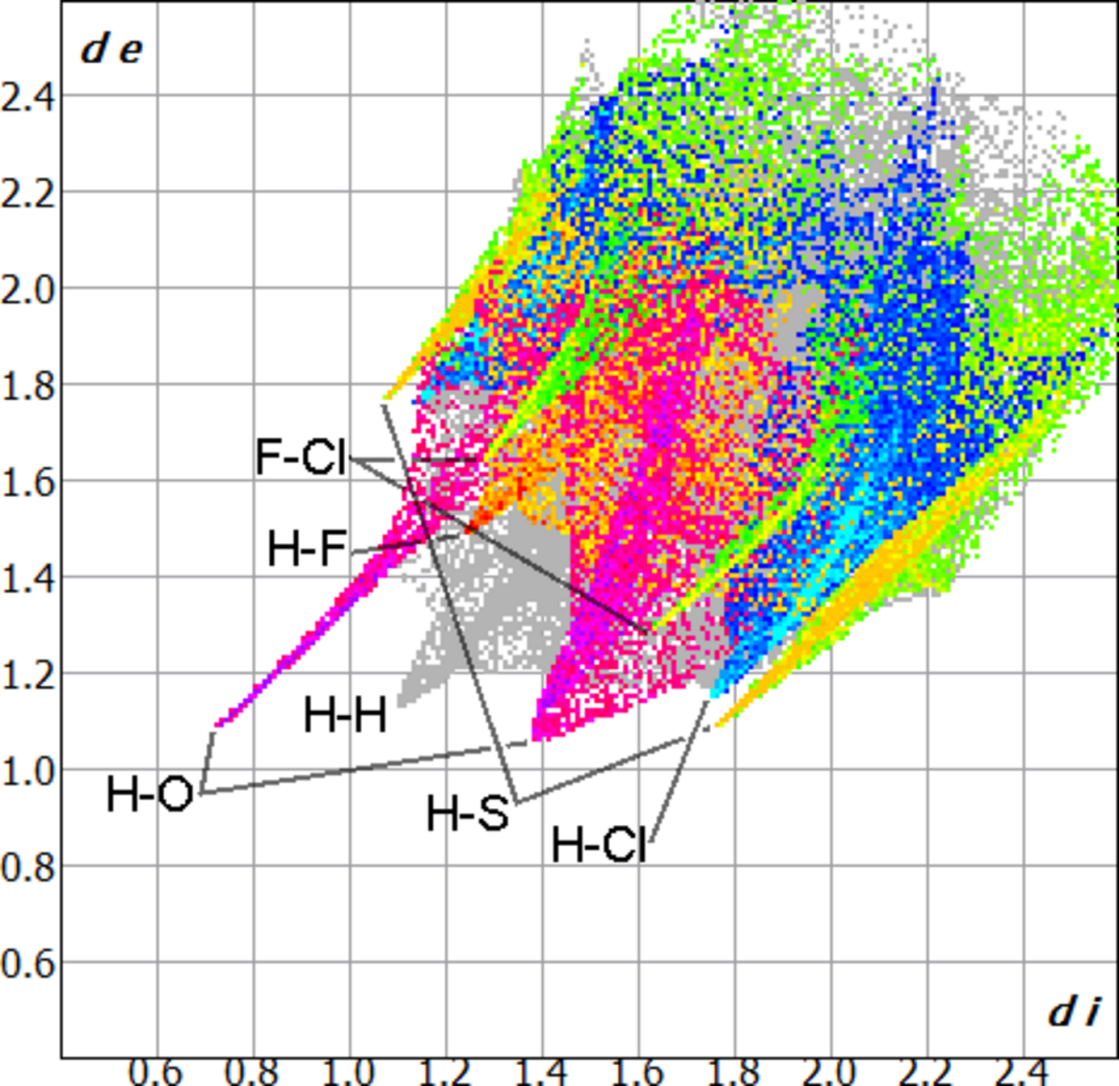
Hirshfeld fingerprint plot of **1** (with DMSO external to the surface) with H⋯O (pink/purple), H⋯F (red/orange), H⋯S (orange), H⋯Cl (blue), and F⋯Cl (yellow/green) contacts superimposed.

Hirshfeld analysis of **1** with the DMSO external to the surface, generated using *CrystalExplorer* (Spackman & Jayatilaka, 2009 ▸; Spackman *et al.*, 2021 ▸), shows sharp peaks in the fingerprint plots for both H⋯O and H⋯S contacts (5.6 and 16.1% of the overall surface, respectively) as would be expected from the observed classical and non-classical hydrogen bonding described above. The H⋯S fingerprint does show a broad tail indicating a diverse range of H⋯S contacts occurring beyond the discrete C—H⋯S hydrogen bonds. Similarly, sharp peaks are observed in the fingerprint plots for both H⋯Cl and H⋯F contacts (11.7 and 5.5% of the overall surface, respectively) consistent with the weaker C—H⋯*X* inter­actions noted above (Fig. 3 ▸). While the fingerprint plot for H⋯H contacts does show a sharp peak, this corresponds to a contact between hydrogen atoms on the DMSO methyl group and an aromatic C—H grouping with H⋯H > 2.3 Å, so it is considered unlikely to represent an attractive inter­action.

## Synthesis and crystallization

**Figure 4:**
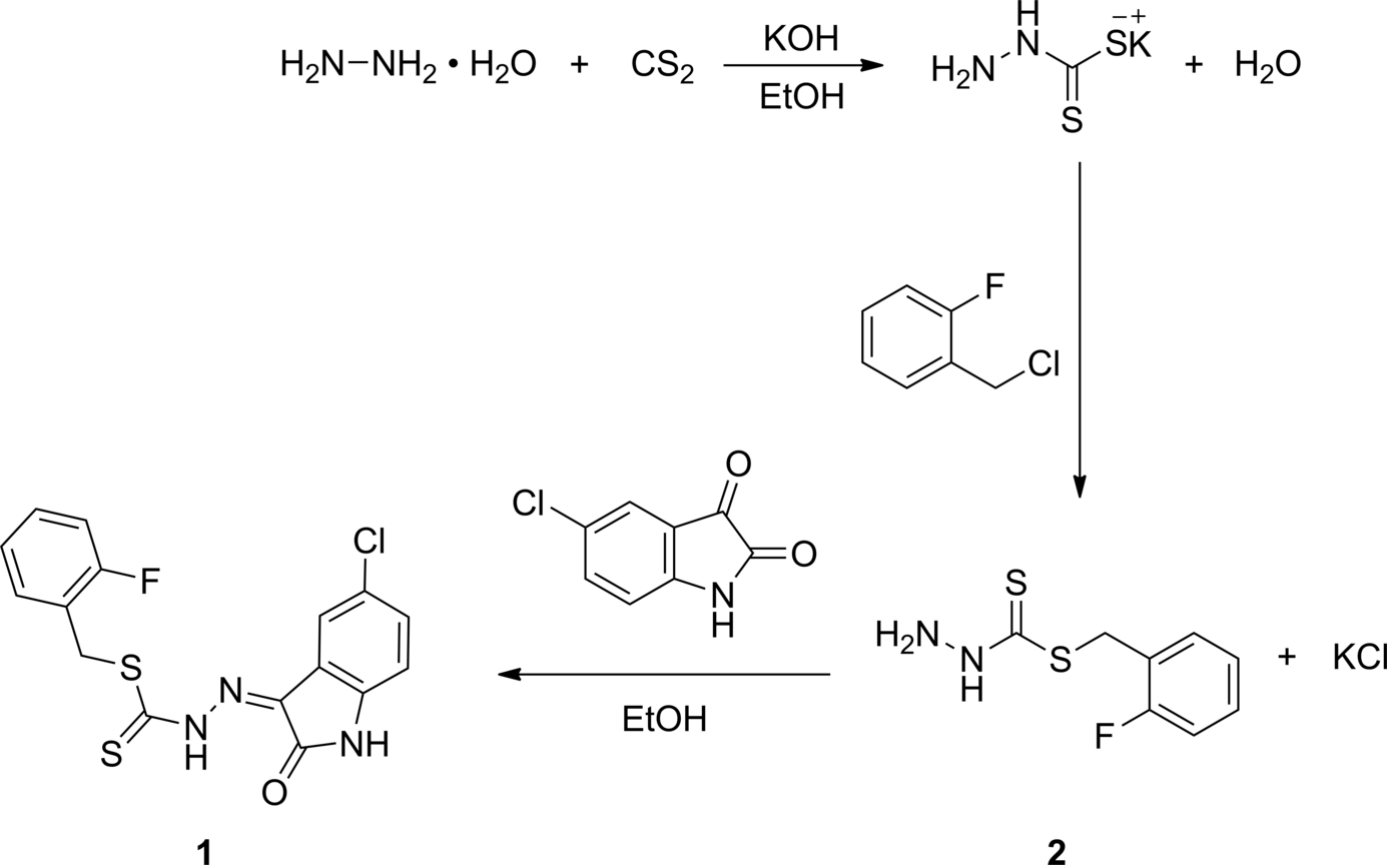
A synthetic scheme for the preparation of **1**.

The 2-fluoro­benzyl hydrazinecarbodi­thio­ate precursor **2**, was synthesized using our published methods for related compounds with minor modifications (Manan *et al.*, 2011 ▸) (Fig. 4 ▸). Potassium hydroxide (11.2 g, 0.2 mol, 1.0 eq) was dissolved in 70 ml of 90% ethanol and to this solution was added hydrazine hydrate (10.0 g, 0.2 mol, 99%, 1.0 eq) and stirred at 0 °C. To the resultant cooled solution, carbon di­sulfide (15.2 g, 0.2 mol, 1.0 eq) was added dropwise, whilst maintaining the solution below 0 °C with constant stirring. Upon addition of carbon di­sulfide, two layers were formed and the lower, brown, layer was collected. 40% ethanol (60 ml) was added to the brown solution and the resulting mixture was cooled in an ice bath while 2-fluoro­benzyl chloride (28.9 g, 0.2 mol, 1.0 eq) was added dropwise with vigorous stirring. The white product formed was filtered and used directly for the next step without further purification.

A solution of 5-chloro­isatin (1.82 g, 10.0 mmol) in hot ethanol (40 ml) was added to a solution of the di­thio­carbazate precursor **2** (2.16 g, 10.0 mmol, 1.0 e.q) in hot ethanol (40 ml). The mixture was heated (80 °C) with continuous stirring for 15 min and later allowed to cool to room temperature and stand for about 20 min., until a precipitate formed, which was then collected by filtration and dried over silica gel. The crude solids were purified by recrystallization from ethanol solution to yield compound **1** as a light-yellow solid (yield: 3.23 g, 85%). m.p 227–228 °C. Elemental analysis calculated for C_16_H_11_ClFN_3_OS_2_: C, 50.59; H, 2.92; N, 11.06%. Found: C, 50.67; H, 2.89; N, 11.01%. FT–IR (KBr, *ν*, cm^−1^): 3155 (NH), 1692 (C=O); 1613 (C=N); 1070 (C=S); 1141 (N—N); ^1^H NMR (400 MHz, *d_6_*-DMSO) δ: (p.p.m.): 4.56 (*s*, 2H), 6.96 (*d*, *J* = 8.3 Hz, 1H) 7.18–7.26 (*m*, 2H), 7.36–7.45 (*m*, 2H), 7.53–7.58 (*m*, 2H), 11.47 (*s*, 1H), 13.89 (*s*, 1H). Crystals suitable for X-ray diffraction were grown by slow evaporation of a dimethyl sulfoxide solution at room temperature.

## Refinement

Crystal data, data collection, and structure refinement details are summarized in Table 2 ▸.

## Supplementary Material

Crystal structure: contains datablock(s) I, general. DOI: 10.1107/S2414314625008491/hb4538sup1.cif

Structure factors: contains datablock(s) I. DOI: 10.1107/S2414314625008491/hb4538Isup2.hkl

Supporting information file. DOI: 10.1107/S2414314625008491/hb4538Isup3.cml

CCDC reference: 2491844

Additional supporting information:  crystallographic information; 3D view; checkCIF report

## Figures and Tables

**Table 1:** Hydrogen-bond geometry (Å, °)

*D*—H⋯*A*	*D*—H	H⋯*A*	*D*⋯*A*	*D*—H⋯*A*
N1—H1⋯O21	0.97 (2)	1.86 (2)	2.822 (4)	169 (5)
N4—H4⋯O2	0.96 (2)	1.98 (3)	2.749 (4)	135 (4)
C7—H7⋯S10^i^	0.95	2.99	3.933 (4)	170

Symmetry code: (i) 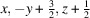.

**Table 2:** Experimental details

Crystal data	
Chemical formula	C_16_H_11_ClFN_3_OS_2_·C_2_H_6_OS
*M*_r_	457.97
Crystal system, space group	Monoclinic, *P*2_1_/*c*
Temperature (K)	100
*a*, *b*, *c* (Å)	22.7322 (9), 4.72526 (18), 18.8781 (6)
β (°)	95.690 (3)
*V* (Å^3^)	2017.81 (13)
*Z*	4
Radiation type	Mo *K*α
μ (mm^−1^)	0.53
Crystal size (mm)	0.35 × 0.02 × 0.01

Data collection	
Diffractometer	Rigaku XtaLAB P200K
Absorption correction	Multi-scan (*CrysAlis PRO*; (Rigaku OD, 2023 ▸)
*T*_min_, *T*_max_	0.324, 1.000
No. of measured, independent and observed [*I* > 2σ(*I*)] reflections	31534, 4903, 2962
*R*_int_	0.132
(sin θ/λ)_max_ (Å^−1^)	0.687

Refinement	
*R*[*F*^2^ > 2σ(*F*^2^)], *wR*(*F*^2^), *S*	0.072, 0.149, 1.02
No. of reflections	4903
No. of parameters	263
No. of restraints	2
H-atom treatment	H atoms treated by a mixture of independent and constrained refinement
Δρ_max_, Δρ_min_ (e Å^−3^)	0.67, −0.61

Computer programs: *CrysAlis PRO* (Rigaku OD, 2023 ▸), *SHELXT2018/2* (Sheldrick, 2015*a* ▸), *SHELXL2019/3* (Sheldrick, 2015*b* ▸), *Mercury* (Macrae *et al.*, 2020 ▸), *enCIFer* (Allen *et al.*, 2004 ▸), *publCIF* (Westrip, 2010 ▸) and *OLEX2* (Dolomanov *et al.*, 2009 ▸).

## full crystallographic data

### 2-Fluorobenzyl (Z)-2-(5-chloro-2-oxoindolin-3-ylidene)hydrazine-1-carbodithioate dimethyl sulfoxide monosolvate Crystal data

**Table:**

C_16_H_11_ClFN_3_OS_2_·C_2_H_6_OS	*F*(000) = 944
*M**_r_* = 457.97	*D*_x_ = 1.508 Mg m^−^^3^
Monoclinic, *P*2_1_/*c*	Mo *K*α radiation, λ = 0.71073 Å
*a* = 22.7322 (9) Å	Cell parameters from 8236 reflections
*b* = 4.72526 (18) Å	θ = 2.3–28.7°
*c* = 18.8781 (6) Å	µ = 0.53 mm^−^^1^
β = 95.690 (3)°	*T* = 100 K
*V* = 2017.81 (13) Å^3^	Needle, yellow
*Z* = 4	0.35 × 0.02 × 0.01 mm

### 2-Fluorobenzyl (Z)-2-(5-chloro-2-oxoindolin-3-ylidene)hydrazine-1-carbodithioate dimethyl sulfoxide monosolvate Data collection

**Table:**

Rigaku XtaLAB P200K diffractometer	4903 independent reflections
Radiation source: Rotating Anode, Rigaku FR-X	2962 reflections with *I* > 2σ(*I*)
Rigaku Osmic Confocal Optical System monochromator	*R*_int_ = 0.132
Detector resolution: 5.8140 pixels mm^-1^	θ_max_ = 29.2°, θ_min_ = 2.3°
shutterless scans	*h* = −31→30
Absorption correction: multi-scan (CrysAlis PRO; (Rigaku OD, 2023)	*k* = −6→6
*T*_min_ = 0.324, *T*_max_ = 1.000	*l* = −24→25
31534 measured reflections	

### 2-Fluorobenzyl (Z)-2-(5-chloro-2-oxoindolin-3-ylidene)hydrazine-1-carbodithioate dimethyl sulfoxide monosolvate Refinement

**Table:**

Refinement on *F*^2^	Primary atom site location: dual
Least-squares matrix: full	Hydrogen site location: mixed
*R*[*F*^2^ > 2σ(*F*^2^)] = 0.072	H atoms treated by a mixture of independent and constrained refinement
*wR*(*F*^2^) = 0.149	*w* = 1/[σ^2^(*F*_o_^2^) + (0.0512*P*)^2^ + 4.1991*P*] where *P* = (*F*_o_^2^ + 2*F*_c_^2^)/3
*S* = 1.02	(Δ/σ)_max_ < 0.001
4903 reflections	Δρ_max_ = 0.67 e Å^−^^3^
263 parameters	Δρ_min_ = −0.61 e Å^−^^3^
2 restraints	

### 2-Fluorobenzyl (Z)-2-(5-chloro-2-oxoindolin-3-ylidene)hydrazine-1-carbodithioate dimethyl sulfoxide monosolvate Special details

**Table:**

Geometry. All esds (except the esd in the dihedral angle between two l.s. planes) are estimated using the full covariance matrix. The cell esds are taken into account individually in the estimation of esds in distances, angles and torsion angles; correlations between esds in cell parameters are only used when they are defined by crystal symmetry. An approximate (isotropic) treatment of cell esds is used for estimating esds involving l.s. planes.
Refinement. Hydrogen atoms on N1 and N4 were located from the F_map_ and refined isotropically with N—H distance restrained. The DMSO solvate in the asymmetric unit is positioned outside the cell to clearly show discrete hydrogen bonding interaction between O21 and N1 The N-bound H atoms were located in a difference map and refined isotropically subject to a distance restraint. The C-bound H atoms were located geometrically (C—H = 0.95–0.99 Å) and refined as riding atoms. The constraint *U*_iso_(H) = 1.2*U*_eq_(phenyl or methylene C) or 1.5*U*_eq_(methyl C) was applied in all cases.

### 2-Fluorobenzyl (Z)-2-(5-chloro-2-oxoindolin-3-ylidene)hydrazine-1-carbodithioate dimethyl sulfoxide monosolvate Fractional atomic coordinates and isotropic or equivalent isotropic displacement parameters (Å^2^)

**Table:**

	*x*	*y*	*z*	*U*_iso_*/*U*_eq_	
Cl6	0.16950 (4)	1.1289 (2)	0.46870 (5)	0.0295 (3)	
S10	0.27118 (4)	−0.1842 (2)	0.11690 (5)	0.0227 (2)	
S11	0.17063 (4)	0.0822 (2)	0.18841 (5)	0.0221 (2)	
F13	0.06348 (11)	0.2730 (6)	0.04236 (12)	0.0380 (6)	
O2	0.39363 (11)	0.3792 (6)	0.26762 (12)	0.0207 (6)	
N1	0.38425 (13)	0.7436 (7)	0.34837 (15)	0.0189 (7)	
H1	0.4256 (11)	0.791 (12)	0.361 (3)	0.073 (18)*	
N3	0.26014 (13)	0.3703 (7)	0.26647 (14)	0.0186 (7)	
N4	0.28239 (13)	0.1886 (7)	0.22016 (15)	0.0178 (7)	
H4	0.3246 (9)	0.180 (10)	0.218 (2)	0.043 (13)*	
C2	0.36404 (15)	0.5343 (8)	0.30279 (18)	0.0169 (8)	
C3	0.29790 (16)	0.5261 (8)	0.30428 (18)	0.0166 (8)	
C5	0.23135 (16)	0.8183 (8)	0.38204 (18)	0.0202 (8)	
H5	0.194712	0.734549	0.364798	0.024*	
C6	0.23440 (16)	1.0262 (9)	0.43396 (18)	0.0228 (9)	
C7	0.28730 (17)	1.1543 (8)	0.45932 (18)	0.0213 (9)	
H7	0.287629	1.296018	0.495051	0.026*	
C8	0.33970 (17)	1.0761 (8)	0.43270 (18)	0.0204 (8)	
H8	0.376092	1.164404	0.449018	0.025*	
C9	0.33718 (16)	0.8657 (8)	0.38168 (18)	0.0180 (8)	
C4	0.28394 (16)	0.7369 (8)	0.35610 (17)	0.0174 (8)	
C10	0.24498 (16)	0.0307 (8)	0.17534 (18)	0.0187 (8)	
C11	0.13311 (17)	−0.1415 (9)	0.11935 (19)	0.0243 (9)	
H11A	0.143520	−0.083799	0.071737	0.029*	
H11B	0.144229	−0.342351	0.127333	0.029*	
C12	0.06787 (16)	−0.0994 (9)	0.12501 (19)	0.0220 (9)	
C13	0.03534 (17)	0.1027 (9)	0.08605 (19)	0.0258 (9)	
C14	−0.02408 (18)	0.1457 (10)	0.0900 (2)	0.0325 (10)	
H14	−0.045051	0.285628	0.061553	0.039*	
C15	−0.05252 (19)	−0.0197 (10)	0.1365 (2)	0.0366 (11)	
H15	−0.093438	0.007759	0.140623	0.044*	
C16	−0.0218 (2)	−0.2235 (10)	0.1766 (2)	0.0368 (11)	
H16	−0.041683	−0.337315	0.208217	0.044*	
C17	0.03795 (18)	−0.2645 (9)	0.1714 (2)	0.0299 (10)	
H17	0.058766	−0.405945	0.199507	0.036*	
S21	0.50398 (4)	1.2715 (2)	0.39061 (4)	0.0183 (2)	
O21	0.49915 (11)	0.9503 (6)	0.38890 (13)	0.0223 (6)	
C21	0.56233 (17)	1.3495 (9)	0.45825 (18)	0.0247 (9)	
H21A	0.550478	1.293115	0.504730	0.037*	
H21B	0.570580	1.553093	0.458431	0.037*	
H21C	0.597955	1.245142	0.448706	0.037*	
C22	0.54195 (17)	1.3726 (9)	0.31624 (18)	0.0220 (9)	
H22A	0.578537	1.262801	0.316345	0.033*	
H22B	0.551476	1.574700	0.319444	0.033*	
H22C	0.516682	1.335640	0.272074	0.033*	

### 2-Fluorobenzyl (Z)-2-(5-chloro-2-oxoindolin-3-ylidene)hydrazine-1-carbodithioate dimethyl sulfoxide monosolvate Atomic displacement parameters (Å^2^)

**Table:**

	*U*^11^	*U*^22^	*U*^33^	*U*^12^	*U*^13^	*U*^23^
Cl6	0.0265 (5)	0.0366 (6)	0.0274 (5)	0.0067 (5)	0.0128 (4)	−0.0034 (5)
S10	0.0261 (5)	0.0255 (6)	0.0178 (4)	0.0005 (4)	0.0079 (4)	−0.0039 (4)
S11	0.0210 (5)	0.0266 (6)	0.0192 (4)	−0.0012 (4)	0.0048 (4)	−0.0054 (4)
F13	0.0392 (14)	0.0445 (16)	0.0311 (13)	0.0032 (13)	0.0083 (11)	0.0046 (12)
O2	0.0227 (14)	0.0226 (15)	0.0181 (12)	0.0011 (12)	0.0090 (11)	−0.0017 (11)
N1	0.0170 (16)	0.0208 (18)	0.0196 (15)	−0.0008 (14)	0.0050 (13)	−0.0001 (14)
N3	0.0243 (17)	0.0199 (18)	0.0127 (14)	0.0041 (14)	0.0070 (12)	0.0024 (13)
N4	0.0189 (16)	0.0200 (18)	0.0153 (14)	−0.0009 (14)	0.0051 (12)	−0.0022 (13)
C2	0.0185 (18)	0.019 (2)	0.0137 (16)	−0.0007 (16)	0.0042 (14)	0.0047 (15)
C3	0.0180 (18)	0.018 (2)	0.0138 (16)	−0.0020 (16)	0.0038 (14)	0.0029 (15)
C5	0.0192 (19)	0.025 (2)	0.0162 (17)	0.0000 (17)	0.0031 (14)	0.0034 (16)
C6	0.023 (2)	0.032 (2)	0.0154 (17)	0.0073 (18)	0.0087 (15)	0.0057 (17)
C7	0.029 (2)	0.019 (2)	0.0164 (17)	0.0038 (17)	0.0059 (16)	−0.0003 (16)
C8	0.023 (2)	0.020 (2)	0.0173 (17)	−0.0012 (17)	0.0002 (15)	−0.0002 (16)
C9	0.0196 (18)	0.020 (2)	0.0155 (16)	0.0027 (16)	0.0048 (14)	0.0047 (15)
C4	0.0219 (19)	0.019 (2)	0.0121 (16)	0.0009 (17)	0.0030 (14)	0.0009 (16)
C10	0.0205 (19)	0.021 (2)	0.0147 (17)	0.0006 (17)	0.0033 (15)	0.0039 (16)
C11	0.026 (2)	0.027 (2)	0.0210 (18)	−0.0011 (18)	0.0052 (16)	−0.0052 (18)
C12	0.0223 (19)	0.026 (2)	0.0174 (17)	−0.0011 (18)	0.0023 (15)	−0.0068 (17)
C13	0.027 (2)	0.031 (2)	0.0196 (18)	−0.0062 (19)	0.0051 (16)	−0.0062 (18)
C14	0.026 (2)	0.039 (3)	0.031 (2)	0.004 (2)	−0.0011 (18)	−0.012 (2)
C15	0.023 (2)	0.043 (3)	0.045 (3)	−0.005 (2)	0.009 (2)	−0.019 (2)
C16	0.037 (3)	0.034 (3)	0.043 (3)	−0.005 (2)	0.020 (2)	−0.010 (2)
C17	0.034 (2)	0.028 (2)	0.029 (2)	−0.003 (2)	0.0106 (18)	−0.0062 (19)
S21	0.0184 (5)	0.0213 (5)	0.0159 (4)	0.0001 (4)	0.0058 (4)	−0.0007 (4)
O21	0.0230 (14)	0.0213 (15)	0.0232 (13)	−0.0031 (12)	0.0057 (11)	−0.0011 (12)
C21	0.026 (2)	0.032 (3)	0.0156 (17)	−0.0019 (19)	0.0012 (16)	−0.0007 (17)
C22	0.026 (2)	0.027 (2)	0.0153 (17)	−0.0025 (18)	0.0092 (15)	−0.0015 (17)

### 2-Fluorobenzyl (Z)-2-(5-chloro-2-oxoindolin-3-ylidene)hydrazine-1-carbodithioate dimethyl sulfoxide monosolvate Geometric parameters (Å, º)

**Table:**

Cl6—C6	1.742 (4)	C9—C4	1.397 (5)
S10—C10	1.653 (4)	C11—H11A	0.9900
S11—C10	1.749 (4)	C11—H11B	0.9900
S11—C11	1.823 (4)	C11—C12	1.511 (5)
F13—C13	1.357 (4)	C12—C13	1.376 (6)
O2—C2	1.232 (4)	C12—C17	1.398 (5)
N1—H1	0.974 (19)	C13—C14	1.375 (5)
N1—C2	1.360 (5)	C14—H14	0.9500
N1—C9	1.417 (4)	C14—C15	1.382 (6)
N3—N4	1.358 (4)	C15—H15	0.9500
N3—C3	1.291 (5)	C15—C16	1.373 (7)
N4—H4	0.964 (19)	C16—H16	0.9500
N4—C10	1.362 (5)	C16—C17	1.385 (6)
C2—C3	1.507 (5)	C17—H17	0.9500
C3—C4	1.453 (5)	S21—O21	1.522 (3)
C5—H5	0.9500	S21—C21	1.786 (4)
C5—C6	1.384 (5)	S21—C22	1.785 (3)
C5—C4	1.390 (5)	C21—H21A	0.9800
C6—C7	1.388 (5)	C21—H21B	0.9800
C7—H7	0.9500	C21—H21C	0.9800
C7—C8	1.388 (5)	C22—H22A	0.9800
C8—H8	0.9500	C22—H22B	0.9800
C8—C9	1.381 (5)	C22—H22C	0.9800
			
C10—S11—C11	102.02 (17)	H11A—C11—H11B	108.8
C2—N1—H1	126 (3)	C12—C11—S11	105.4 (3)
C2—N1—C9	110.8 (3)	C12—C11—H11A	110.7
C9—N1—H1	123 (3)	C12—C11—H11B	110.7
C3—N3—N4	116.5 (3)	C13—C12—C11	122.2 (4)
N3—N4—H4	119 (3)	C13—C12—C17	116.9 (4)
N3—N4—C10	119.8 (3)	C17—C12—C11	120.9 (4)
C10—N4—H4	121 (3)	F13—C13—C12	118.4 (3)
O2—C2—N1	127.2 (3)	F13—C13—C14	118.1 (4)
O2—C2—C3	126.4 (3)	C14—C13—C12	123.5 (4)
N1—C2—C3	106.4 (3)	C13—C14—H14	120.8
N3—C3—C2	127.9 (3)	C13—C14—C15	118.4 (4)
N3—C3—C4	125.8 (3)	C15—C14—H14	120.8
C4—C3—C2	106.3 (3)	C14—C15—H15	119.9
C6—C5—H5	121.3	C16—C15—C14	120.2 (4)
C6—C5—C4	117.5 (3)	C16—C15—H15	119.9
C4—C5—H5	121.3	C15—C16—H16	119.8
C5—C6—Cl6	118.6 (3)	C15—C16—C17	120.5 (4)
C5—C6—C7	122.3 (3)	C17—C16—H16	119.8
C7—C6—Cl6	119.1 (3)	C12—C17—H17	119.7
C6—C7—H7	119.9	C16—C17—C12	120.5 (4)
C8—C7—C6	120.3 (3)	C16—C17—H17	119.7
C8—C7—H7	119.9	O21—S21—C21	105.54 (18)
C7—C8—H8	121.1	O21—S21—C22	106.83 (17)
C9—C8—C7	117.8 (4)	C22—S21—C21	97.15 (18)
C9—C8—H8	121.1	S21—C21—H21A	109.5
C8—C9—N1	128.4 (3)	S21—C21—H21B	109.5
C8—C9—C4	122.0 (3)	S21—C21—H21C	109.5
C4—C9—N1	109.6 (3)	H21A—C21—H21B	109.5
C5—C4—C3	132.8 (3)	H21A—C21—H21C	109.5
C5—C4—C9	120.2 (3)	H21B—C21—H21C	109.5
C9—C4—C3	107.0 (3)	S21—C22—H22A	109.5
S10—C10—S11	126.6 (2)	S21—C22—H22B	109.5
N4—C10—S10	120.5 (3)	S21—C22—H22C	109.5
N4—C10—S11	112.9 (3)	H22A—C22—H22B	109.5
S11—C11—H11A	110.7	H22A—C22—H22C	109.5
S11—C11—H11B	110.7	H22B—C22—H22C	109.5
			
Cl6—C6—C7—C8	179.9 (3)	C6—C5—C4—C9	−0.8 (5)
S11—C11—C12—C13	91.9 (4)	C6—C7—C8—C9	−1.1 (5)
S11—C11—C12—C17	−87.3 (4)	C7—C8—C9—N1	−177.7 (3)
F13—C13—C14—C15	−177.5 (4)	C7—C8—C9—C4	1.2 (5)
O2—C2—C3—N3	−4.6 (6)	C8—C9—C4—C3	−179.4 (3)
O2—C2—C3—C4	176.9 (3)	C8—C9—C4—C5	−0.2 (5)
N1—C2—C3—N3	176.1 (3)	C9—N1—C2—O2	−177.0 (3)
N1—C2—C3—C4	−2.4 (4)	C9—N1—C2—C3	2.3 (4)
N1—C9—C4—C3	−0.3 (4)	C4—C5—C6—Cl6	−178.9 (3)
N1—C9—C4—C5	178.8 (3)	C4—C5—C6—C7	0.9 (5)
N3—N4—C10—S10	178.1 (3)	C10—S11—C11—C12	−177.7 (3)
N3—N4—C10—S11	−2.5 (4)	C11—S11—C10—S10	−3.0 (3)
N3—C3—C4—C5	4.1 (6)	C11—S11—C10—N4	177.6 (3)
N3—C3—C4—C9	−176.9 (3)	C11—C12—C13—F13	−1.4 (5)
N4—N3—C3—C2	0.8 (5)	C11—C12—C13—C14	179.9 (4)
N4—N3—C3—C4	179.0 (3)	C11—C12—C17—C16	179.6 (4)
C2—N1—C9—C8	177.7 (4)	C12—C13—C14—C15	1.1 (6)
C2—N1—C9—C4	−1.3 (4)	C13—C12—C17—C16	0.4 (6)
C2—C3—C4—C5	−177.4 (4)	C13—C14—C15—C16	−0.8 (6)
C2—C3—C4—C9	1.6 (4)	C14—C15—C16—C17	0.4 (7)
C3—N3—N4—C10	−175.8 (3)	C15—C16—C17—C12	−0.2 (6)
C5—C6—C7—C8	0.1 (6)	C17—C12—C13—F13	177.8 (3)
C6—C5—C4—C3	178.1 (4)	C17—C12—C13—C14	−0.8 (6)

### 2-Fluorobenzyl (Z)-2-(5-chloro-2-oxoindolin-3-ylidene)hydrazine-1-carbodithioate dimethyl sulfoxide monosolvate Hydrogen-bond geometry (Å, º)

**Table:**

*D*—H···*A*	*D*—H	H···*A*	*D*···*A*	*D*—H···*A*
N1—H1···O21	0.97 (2)	1.86 (2)	2.822 (4)	169 (5)
N4—H4···O2	0.96 (2)	1.98 (3)	2.749 (4)	135 (4)
C7—H7···S10^i^	0.95	2.99	3.933 (4)	170

Symmetry code: (i) *x*, −*y*+3/2, *z*+1/2.
